# 核酸干扰药物在抗肿瘤领域的机遇和挑战

**DOI:** 10.3779/j.issn.1009-3419.2022.102.20

**Published:** 2022-07-20

**Authors:** 锐 陈, 燕 徐

**Affiliations:** 1 100730 北京，中国医学科学院北京协和医院临床药理研究中心 Clinical Pharmacology Research Center, Peking Union Medical College Hospital, Chinese Academy of Medical Science, Beijing 100730, China; 2 100730 北京，中国医学科学院北京协和医院呼吸与危重症医学科 Department of Respiratory and Critical Care Medicine, Peking Union Medical College Hospital, Chinese Academy of Medical Science, Beijing 100730, China

**Keywords:** 核酸干扰, 肿瘤, 递送系统, RNA interference, Oncology, Delivery systems

## Abstract

随着核酸干扰机制的发现及一系列技术难题的逐步攻克，非肿瘤领域的多款核酸干扰药物在国外获批上市，其以特异性高、疗效持续时间长、研发成功率高等优势，成为令全球瞩目的新兴领域。在肿瘤领域尚未有此类新药上市，人们对这一领域的突破寄予厚望。本文阐述核酸干扰药物的特点、潜在抗肿瘤机制、递送系统的难点及在抗肿瘤领域的进展，分析其在非肿瘤领域的成功难以在肿瘤领域被简单复制的潜在原因，为研发及临床转化提供参考。

## Abstract (en)

As the discovery of RNA interference (RNAi) and the gradual conquering of a series of technical issues, a few of RNAi therapeutics have been approved in the non-tumor field abroad. With the advantages of high specificity, long duration of efficacy, and high success rate of development, RNAi therapeutics have become the emerging field globally. There are no RNAi therapeutics approved in oncology so far, and people are hoping a breakthrough in the field. In the present article, the characteristics and potential anti-tumor mechanism of RNAi therapeutics, difficulties in delivery system and progress in oncology are described, and the potential reasons why their success in non-tumor field is difficult to be simply replicated in tumor field are analyzed, providing reference for research and clinical transformation of RNAi therapeutics in oncology.

1998年，科学家Andrew Fire和Craig Mello首次提出会导致基因沉默的RNA干扰机制。8年后，两人凭借这一发现获得2006年度诺贝尔生理学或医学奖，认为其发现了“控制遗传信息流动的基本机制”^[1]^。这一机制孕育而生的核酸干扰药物的研究进展飞速，近5年，10款核酸干扰药物在美国食品药品监督管理局（Food and Drug Administration, FDA）和欧洲药品管理局（European Medicines Agency, EMEA）获批，众多产品在研发中。在过去20年间，核酸干扰药物的成药性差、脱靶毒性、递送系统等一系列技术难题被逐步攻克，全球迎来核酸干扰药物的研发热潮^[2]^。核酸干扰药物在肿瘤领域尚未有新药获批上市，但众多产品在研，人们对这一领域的突破寄予厚望。

## 1 RNAi机制及核酸干扰药物潜在抗肿瘤机制

1998年，Andrew Fire和Craig Mello发现双链RNA是秀丽隐杆线虫转录后基因沉默的致病因子，并将这种现象称为RNA干扰（RNA interference, RNAi）。RNAi的发现解释了植物和真菌中令人费解的基因沉默现象，并带来生物学变革，表明非编码RNA是基因表达的核心调控因子^[3]^。人类基因组中98.5%的序列为非蛋白质编码区，这部分既往被认为是无功能片段，研究^[4, 5]^表明，大约80%的基因组都具有生物活性并被转录为RNA，而不被翻译为蛋白质。这些非编码RNA（non-coding RNA, ncRNA）虽不翻译蛋白质，但可通过RNAi机制调节其他基因的转录和翻译。在肿瘤的发生发展中，ncRNA扮演着重要的角色，因此通过RNAi机制调控肿瘤生长成为新药研发的重要靶点^[6, 7]^。RNAi调节基因表达的潜在机制包括：①核糖核酸酶H（RNase H）介导的mRNA降解；②RNA诱导沉默复合体（RNA-induced silencing complex, RISC）介导的mRNA降解；③调节mRNA的剪接；④抑制或增强mRNA的转译；⑤与微RNA竞争等^[8]^。

核酸干扰药物可以调控肿瘤细胞在增殖、分化、凋亡、血管生成、转移和耐药等关键过程的转录翻译，成为治疗肿瘤的潜在疗法。如已证实ncRNA在肺癌中具有多种调控作用，参与诱导增殖、抑制凋亡、增加癌细胞转移潜能和耐药性获得等方面，肿瘤细胞中多种ncRNA失调是未来治疗的靶点^[9]^。

## 2 核酸干扰药物的特点

核酸干扰药物主要包括反义寡核苷酸（antisense oligonucleotide, ASOs）、小干扰RNA（small interfering RNA, siRNA）和微小RNA（micro RNA）等。在人类基因组中，仅1.5%的序列编码蛋白质（疾病相关蛋白占10%-15%），且其中多数不能被目前常规的小分子和生物大分子药物所靶向，属于不可成药靶点，目前已批准的药物仅关联到人类基因组的0.05%^[10]^。RNA靶向疗法可以大幅扩大人类基因组中可用治疗靶点的比例，且研发成功率远高于传统大小分子^[11]^。核酸干扰药物基于疾病基因序列研发，特异性高，疗效持续时间长，开发时间短，生产成本低于生物大分子，这些优势都使得核酸干扰药物成为生物医药新的热点和希望。但与此同时，核酸干扰药物也有一些局限性，其在血液中不稳定，容易被RNA酶降解，主动靶向性差，核酸干扰药物要在细胞内才能发挥作用，而小核酸裸链靠内吞作用进入细胞效率极低，常需要外部的递送系统辅助。

## 3 递送系统的难点和挑战

如前所述，小核酸在血液中不稳定，裸链进入肿瘤细胞的效率极低，因此递送系统的开发成为核酸干扰药物研发的关键环节。理想的递送系统应具备以下特点：①具有生物相容性、生物可降解性和非免疫原性；②在通过循环运输和释放到内涵体的过程中保护RNA不受血清核酸酶的降解；③避免被肝脏或肾脏快速清除；④递送RNA药物进入靶细胞，同时不影响正常组织^[1]^。

目前开发的用于核酸干扰药物尤其是siRNA药物的递送系统主要包括：①化学修饰；②脂质纳米颗粒递送系统；③聚合物递送系统；④共价结合递送系统；⑤核酸干扰药物和小分子共同递送；⑥病毒载体等^[1, 3, 12]^。这些递送系统有助于增加小核酸的血浆稳定性，减少细胞毒性，靶向递送到肿瘤组织，且递送系统自身可清除。若递送系统设计不佳，可能带来额外问题，如脂质纳米颗粒表面大量的正电荷可能导致红细胞聚集，人工合成的大分子量递送系统可能难以降解从而导致毒性等。

2018年，全球第一个siRNA药物Patisiran获FDA批准上市，Patisiran使用脂质纳米颗粒递送系统，siRNA被包裹在脂质纳米颗粒（lipid nanoparticle, LNP）中，递送到靶组织。但Patisiran的脂质递送系统会引起促炎症反应，在治疗前需要输注皮质类固醇、对乙酰氨基酚和抗组胺药物来预防给药后的炎症反应^[2, 13]^。此后FDA批准了3个siRNA药物（Givosiran, Lumasiran, Inclisiran）都是基于siRNA-GalNAc共价结合递送系统，GalNAc可以被肝细胞表面去唾液酸糖蛋白受体（asialoglycoprotein receptor, ASGPR）识别，并将其摄取进入靶向的肝细胞中^[2]^。GalNAc只能靶向肝脏，对于全身各部位的肿瘤，越来越多递送系统被开发出来，目标更轻的炎症反应以及和特异肿瘤组织更强的亲和力，以实现定向递送^[14, 15]^。如根据既往研究，肿瘤组织内部缺氧，发生厌氧反应并产生大量乳酸，导致肿瘤组织局部pH值下降。根据此机制可开发对低pH敏感的脂质递送系统，在低pH环境中，脂质体不稳定，和细胞膜融合，将小核酸释放送入细胞内^[16, 17]^。又如，聚乳酸和聚乙醇酸的共聚物[poly (lactic-co-glycolic acid), PLGA]是核酸干扰药物常用的递送系统，研究人员用壳聚糖修饰PLGA，利用壳聚糖的粘附性，延长药物在膀胱尿路上皮的滞留时间，已用于针对膀胱肿瘤的核酸干扰药物的开发^[18]^。

## 4 核酸干扰药物在抗肿瘤领域的挑战

目前已上市的核酸干扰药物中没有针对肿瘤适应症的，国际上多款针对肿瘤的核酸干扰类药物处于临床研究阶段（表 1）。随着非肿瘤领域核酸干扰药物的陆续获批，人们对肿瘤领域的突破寄予了厚望。理论上肿瘤领域有许多靶点适合核酸干扰药物的开发，经过全球十余年临床研发，有一些药物处于临床Ⅰ期-Ⅲ期。如靶向血管内皮生长因子（vascular endothelial growth factor, VEGF）和纺锤体驱动蛋白（kinesin spindle protein, KSP）的核酸干扰药物ALN-VSP，由LNP包裹着siRNA，在首次人体研究中，用于治疗肝脏转移的肿瘤患者。给药后1周内，对患者的肝脏穿刺活检可观察到siRNA介导的靶点mRNA的切割及表达下调，进而观察到抗肿瘤活性。有1例子宫内膜癌肝转移的患者，在40次给药后，观察到肿瘤完全缓解（complete response, CR），3例肾癌和胰腺神经内分泌肿瘤肝转移的患者多次给药后观察到疾病稳定状态（stable disease, SD）。同时观察到2周1次静脉给予ALN-VSP在人体是安全可耐受的。研究^[19]^结果为核酸干扰药物的抗肿瘤机制提供了临床证据。

**表 1:** 国际上处于临床试验阶段的抗肿瘤核酸干扰药物研发 Research and development of RNA interference therapeutics in oncology in the stage of clinical trials globally

Drug name	Target	Indication	Delivery system	Sponsor	Clinical stage	Clinical trials No.
CALAA-01	RRM2	Advanced solid tumor	Cyclodextrin nanoparticles	Calando	Phase 1	NCT00689065
siG12D LODER	KRAS/G12D	Pancreatic ductal adenocarcinoma, pancreatic cancer	Polymeric nanoparticles	Silenceed	Phase 1 Phase 2	NCT01188785 NCT01676259
Atu027	PKN3	Pancreatic cancer	Cationic LNPs	Silence	Phase 1 Phase 1/2	NCT00938574 NCT01808638
siRNA-EphA2-DOPC	EphA2	Advanced solid tumor	Neutral LNPs-liposomes	M.D. Anderson	Phase 1	NCT01591356
DCR-MYC	MYC	Hepatocellular carcinoma, solid tumor, lymphoma, multiple myeloma	LNPs	Dicerna	Phase 1 Phase 1b/2	NCT02110563 NCT02314052
ALN-VSP02	KSP/VEGF	Solid tumor	SNALPs	Alnylam	Phase 1	NCT00882180 NCT01158079
TKM-080301	PLK1	Advanced hepatocellular carcinoma, adrenal cortical carcinoma, colorectal/pancreatic/gastric/breast/ovarian cancer with liver metastasis	LNPs	Arbutus	Phase 1 Phase 2	NCT02191878 NCT01262235 NCT01437007
KRAS G12D siRNA	KRAS/G12D	Pancreatic ductal adenocarcinoma, pancreatic cancer	Exosome	M.D. Anderson	Phase 1	NCT03608631
APN401	Cbl-b/DC	Pancreatic cancer, colorectal cancer, melanoma, kidney cancer, solid tumor	*Ex vivo* electroporation	Apeiron	Phase 1	NCT02166255 NCT03087591
MRX34	miR-32 mimic	Hepatocellular carcinoma, hematological malignancy, solid tumor, melanoma	NOV340 SMARTICLE LNP	Mirna	Phase 1	NCT01829971 NCT02862145
TKM-080301	PLK1	Neuroendocrine tumor, adrenal cortical tumor	LNP	Arbutus	Phase 1 Phase 2	NCT01262235 NCT02191878
pbi-shRNA STMN	STMN	Metastatic cancer	LNP	Gradalis	Phase 1	NCT01505153
ARO-HIF2	HIF-2*α*	Renal clear cell carcinoma	TRiM	Arrowhead	Phase 1	NCT04169711
ION537	YAP1	Advanced solid tumor	LICA	Inois	Phase 1	NCT04659096
IONIS-AR-2.5Rx	AR	Prostate cancer	LICA	Inois	Phase 1/2	NCT03300505
IONIS-STAT3Rx	STAT3	Advanced tumor, diffuse large B-cell lymphoma, lymphoma	LICA	Inois	Phase 1/2	NCT01563302
Cobomarsen	microRNA-155	Cutaneous T-cell lymphoma, granuloma fungoides, chronic lymphocytic leukemia, diffuse large B-cell lymphoma, adult T-cell leukemia/lymphoma	NA	Viridian	Phase 2	NCT03837457 NCT03713320 NCT02580552
STP705	TGF-*β*1/COX-2	Basal cell carcinoma, hepatocellular carcinoma, hepatic metastasis, cholangiocarcinoma, cutaneous squamous cell carcinoma *in situ*	Polymeric nanoparticles	Sirnaomics	Phase 1 Phase 2	NCT04669808 NCT04293679 NCT04676633 NCT04844983
STP707	TGF-*β*1/COX-2	Solid tumor	Polymeric nanoparticles	Sirnaomics	Phase 1	NCT05037149
INT-1B3	miR-193a-3p	Solid tumor	LNP	InteRNA	Phase 1	NCT04675996
RX-0201	AKT-1	Renal cell carcinoma, pancreatic cancer	Lipid-coated albumin nanoparticle	Ocuphire	Phase 1 Phase 2	NCT01028495 NCT02089334
TargomiRs	miR-16 mimic	Malignant pleural mesothelioma, non-small cell lung cancer	EGFR-antibody targeted minicells	EnGeneIC	Phase 1	NCT02369198
LNP: lipid nanoparticles; SNALP: stable nucleic acid-lipid particles; TRiM: target RNAi molecule; LICA: ligand conjugated antisense; VEGF: vascular endothelial growth factor; HIF-2*α*: hypoxia-inducible factor 2*α*; TGF-*β*1: transforming growth factor *β*1; COX-2: cyclooxygenase 2; EGFR: epidermal growth factor receptor.						

但人们也发现核酸干扰药物抗肿瘤的研发比人们预期的难度更大。核酸干扰药物在抗肿瘤领域的挑战可能主要有以下几点：①病理机制的异质性。目前已经获批的核酸干扰药物机制明确，主要针对单一致病机制或单一基因，靶向性强，疗效显著。肿瘤的发病机制较复杂，肿瘤内部细胞异质性高，通过抑制单一基因来抑制肿瘤生长疗效有局限性；②开发肿瘤组织特异性递送系统难度较大。核酸干扰类药物对递送系统要求较高，递送系统除需满足前述要求外，对于肿瘤组织的亲和力应远高于正常组织，以降低脱靶效应；③肿瘤细胞对核酸干扰药物摄取率低^[20]^。小核酸在血液中容易被降解，即使到达肿瘤组织，常需通过胞吞作用进入肿瘤细胞，再逃离内涵体进入细胞质中，这两个步骤的效率都较低，提高小核酸在肿瘤细胞内的暴露难度较大。

肿瘤细胞的异质性使得核酸干扰药物在一些单基因遗传病或罕见病上的成功难以被直接复制到抗肿瘤领域。经过多年实践探索，仅1个核酸干扰药物治疗肿瘤进展到Ⅲ期临床试验^[21]^。核酸干扰药物和其他药物联合治疗肿瘤获得了更多关注，可能是研发的方向之一。在肿瘤辅助治疗、耐药治疗方面，核酸干扰药物可能发挥的潜在作用也值得进一步研究。

## 5 总结与展望

基础生物学的发展孕育了核酸干扰药物的新起，标志着人类对体内基因表达的认识和调控水平有了大幅增长。在一些单一机制或单基因疾病上的成功，让人们更真切认识到核酸干扰药物的巨大潜能。在抗肿瘤治疗领域，经历十多年的临床研发，尚未有核酸干扰药物获批，仅1个候选药物进入Ⅲ期，而有相当一部分在Ⅰ期或Ⅱ期被终止，表明核酸干扰药物在非肿瘤领域的成功难以在肿瘤领域被简单复制。递送系统的开发是核酸干扰药物研发的最大难点，而抗肿瘤领域对此要求更高，此外，肿瘤细胞的异质性也使得抑制单一基因有疗效的局限性。降低脱靶效应、提高肿瘤细胞内的药物暴露以及联合治疗等都是核酸干扰药物在肿瘤领域的攻克方向。核酸干扰药物的作用机制确切，研发优势大，成本较低，随着新的递送系统不断涌现、基础生物学不断发展，这一领域将为我们带来新药研发理念的变革和实践的飞跃。
